# Anakinra for dengue patients with hyperinflammation: protocol for a randomized double-blind placebo-controlled trial

**DOI:** 10.12688/wellcomeopenres.21017.1

**Published:** 2024-11-22

**Authors:** Tran Bang Huyen, Huynh Trung Trieu, Nguyen Lam Vuong, Nguyen Minh Nguyet, Dong Thi Hoai Tam, Angela McBride, Nguyen Thi My Linh, Dang Trong Thuan, Nguyen Thanh Phong, Truong Ngoc Trung, Nguyen Thi Cam Huong, Tran Thi Dong Vien, Huynh Thi Le Duyen, Vo Thi My Hoa, James Watson, Ronald Geskus, Phan Vinh Tho, Evelyne Kestelyn, Phan Tu Qui, Sophie Yacoub

**Affiliations:** 1 Oxford University Clinical Research Unit (OUCRU), Ho Chi Minh City, Outside US & Canada, 720000, Vietnam; 2 Hospital for Tropical Diseases, Ho Chi Minh City, Ho Chi Minh, 70000, Vietnam

**Keywords:** dengue, dengue with warning signs, severe dengue, anakinra, interleukin-1 blockade, IL-1 blockade, IL-1 receptor antagonist, trial

## Abstract

**Background:**

Novel host-directed therapies are urgently needed for patients with dengue, particularly those at high risk of developing severe disease. Broad immunosuppression using corticosteroids in unselected patients with dengue has so far been unsuccessful. Patients with hyperinflammation (raised CRP and/or ferritin levels) are at highest risk of poor outcomes in dengue. Anakinra is a licensed, bio-engineered form of the naturally occurring IL-1R antagonist which has shown efficacy in other acute viral-associated hyperinflammatory syndromes.

**Methods:**

This is a randomized placebo-controlled phase II trial of anakinra in 160 patients ≥ 12 years old, diagnosed as having dengue with warning signs or severe dengue and the hyperinflammatory syndrome (plasma ferritin >2000 ng/ml). Participants will receive a 4-day course of either anakinra or placebo. The primary endpoint is the efficacy of anakinra measured by the delta mSOFA score* (change in mSOFA score over 4 days after randomization). The accompanying immunological and transcriptomic analyses aim to identify novel mechanisms and pathways that may represent future biomarkers and therapeutic targets.

**Discussion:**

The observed immunomodulatory benefit of anakinra in acute viral-associated hyperinflammatory syndromes including COVID-19 and auto-immune diseases makes this medication a promising potential treatment for dengue patients with hyperinflammation. This trial will assess the safety and efficacy of anakinra in patients with severe dengue or at high risk of developing life-threatening dengue disease.

**Registration:**

ClinicalTrials.gov (NCT05611710).

## Plain Language Summary

Dengue is a globally important viral infection spread by mosquitoes. Most patients recover after a week, however a minority can progress to severe dengue with shock (a collapse of the circulatory system) or severe organ damage, which can be life-threatening. There are currently no specific therapies to change the disease course or outcomes. New treatments are urgently needed, especially for those with the most severe form of the disease.

Research has shown that patients who have an excessive inflammatory response—when the body’s immune response to the virus is unregulated—have worse outcomes. Modulating this immune response could be a potential treatment approach. Recent studies suggested that interleukin-1 (IL-1) may play an important role in driving the hyper-inflammation during dengue. Blocking IL-1 could regulate the body’s response and improve clinical outcomes.

In this clinical trial, we aim to investigate whether IL-1 blockade - using an established drug, anakinra, is safe and effective to treat moderate-to-severe dengue patients. Anakinra has already demonstrated safety and efficacy in hyperinflammatory syndromes caused by other viral infections, such as COVID-19.

The study will recruit 160 patients, hospitalised with moderate to severe dengue who exhibit an excessive inflammatory response (indicated by a high level of the inflammatory marker, ferritin). Participants will receive either a 4-day course of anakinra or a placebo (visually matched syringes containing normal saline). Participants will be monitored closely, including their clinical manifestations, laboratory tests, and changes in disease severity over the 4-day treatment period, to assess the treatment's safety and effectiveness. Additionally, we aim to explore immunological and gene expression changes to identify new potential biomarkers and treatments.

In summary, anakinra has demonstrated benefits in regulating immune responses in other hyperinflammatory conditions related to acute viral infections. This study will evaluate the safety and efficacy of anakinra in treating patients with moderate-severe dengue and hyperinflammation.

## Background

Dengue continues to be a major public health burden in Vietnam and globally ^1^. While most clinical cases of dengue resolve after 1 week, approximately 5–10% of hospitalized patients will develop severe manifestations, usually during the critical phase (day 4–6 of illness) ^2^, allowing a window of opportunity to identify patients at risk of progression and intervene with a disease modifying agent ^3, 4^. However, thus far, no antiviral agents or adjunctive therapies have been found to alter disease outcomes in dengue ^3– 6^. Novel host directed therapeutics are urgently needed for patients with dengue, particularly those at high risk of developing severe disease.

Understanding of the mechanism underlying progression to severe disease has improved; it has been demonstrated that hypercytokinemia and an excessive host inflammatory response to infection are associated with life-threatening complications ^2, 3^. The dengue virus (DENV) replicates within monocytes and macrophages, and failure to clear these virus-infected cells by dysfunctional cytotoxic NK/CD8+ T cells can facilitate a hyperinflammatory state or “cytokine storm”, through persistent macrophage activation and ultimately end-organ damage. Therapeutic trials with broad immunosuppression using corticosteroids in dengue have so far been unsuccessful ^5^, likely due to unselected patient populations with insufficient stratification. Interleukin-1 (IL-1) is a key pro-inflammatory cytokine involved in the cytokine storm and also in dengue pathogenesis ^7– 9^. One of the safest and possibly most targeted ways of controlling this excessive inflammation is through IL-1 receptor (IL-1R) blockade ^6^.

### Anakinra

Anakinra is a licensed, bio-engineered form of the naturally occurring IL-1R antagonist that blocks the action of IL-1. Clinical trials have shown efficacy of anakinra in reducing adverse outcomes in other acute viral-associated hyperinflammatory syndromes, including COVID-19 ^10– 12^. This drug has already been licensed in various countries and is currently used to treat signs and symptoms of rheumatoid arthritis, cryopyrin-associated periodic syndromes, familial Mediterranean fever, Still’s disease and COVID-19 ^13^.

In dengue, a recent publication reported experience of administering anakinra intravenously, in addition to intravenous methylprednisolone and intravenous immunoglobulins, to a returning traveller with severe dengue and a recent diagnosis of systemic lupus erythematosus, who had clinical, biochemical and bone marrow features consistent with secondary haemophagocytic lymphohistiocytosis (HLH). Initiation of anakinra was followed by resolution of the HLH markers and reduced requirements for multi-organ support ^14^.

### Cytokine storm and inflammatory biomarkers

Previous studies have shown that features of Macrophage activation syndrome (MAS)/secondary HLH/hyperinflammation are more prevalent in severe dengue. Higher inflammatory biomarkers, including higher ferritin ^8, 9, 15^ and an early higher peak plasma IL-1R antagonist (IL-1RA) levels (a surrogate for IL-1 activity) ^7^, are associated with more severe clinical outcomes in dengue. In a cohort of 207 hospitalized dengue patients (children and adults) in Vietnam, 63% with severe disease and 33% with moderate disease had ferritin levels > 2000 ng/ml (Vuong NL, unpublished data). In another cohort of 135 adults with dengue shock in Ho Chi Minh City, median plasma ferritin measured within 24 hours of shock onset was 7671 ng/ml, but among those who did not survive their illness, median plasma ferritin was 71,573 ng/ml at enrolment, and 100,000 ng/ml 48 hours later ^16^.

While measurement of IL-1 activity is complicated due to low concentrations, local production and short half-life ^7^, ferritin testing is readily available in clinical practice. In this trial, we propose using ferritin as a screening biomarker to identify patients at high risk of severe and life-threatening dengue, in whom immunomodulation may improve clinical outcomes.

### Cellular immunology

Cytotoxic NK/CD8+ T cells are involved in the control of macrophage activation and direct killing of virus-infected cells. Dysfunctional NK/CD8+ T cells underlie hyper-inflammatory phenotypes; resulting in uncontrolled macrophage expansion and activation that augments and prolongs the release of pro-inflammatory cytokines. NK cell cytotoxic ability correlates with effective dengue viral clearance and better clinical outcomes ^17^. In CD8+ T cells, selective loss of cytotoxic function is proposed to cause an imbalance in the negative feedback loop of antigen presentation and cytokine secretion. The subsequent inappropriate expansion of this cell type leads to excessive production of T-cell–derived cytokines, resulting in histologic and clinical features of HLH ^18^. We have shown that patients with severe dengue have a lower percentage of activated and proliferating NK cells and lower expression of the cytotoxic granules perforin and granzyme B when compared to patients with less severe dengue infection ^19^. 

Monocytes and macrophages are the main target cell for dengue viral replication and a major source of inflammatory cytokines. Macrophages are involved in all stages of the inflammatory process, polarized by their environment to have inflammatory initiation and propagation (M1) or resolution (M2) or mixed functions. An imbalance of M1/M2 macrophages may induce pathological consequences, from excessive inflammatory mediators or an inadequate production of resolution mediators ^20^. The association between macrophage polarization phenotypes and disease outcome in dengue, however, is largely unknown. We hypothesize that switching off the cytokine-mediated hyperinflammatory loop with IL-1R blockade will restore T/NK cell and M1/M2 homeostasis and prevent downstream tissue damage.

### Genetic associations

Genetic polymorphisms may alter the NK cell cytotoxicity pathway and create hyperinflammatory phenotypes upon immune activation, triggered by various infections. Some of these genes, including those encoding perforin and proteins required for movement and fusion of perforin-containing granules, are well described in MAS ^21^. Further support for a possible key role of NK cells is provided by a genome-wide association study by our group that identified an association between a polymorphism in MICB (an NK cell-activating ligand) and dengue severity ^22^. We assessed the association of these known and novel NK cell single nucleotide polymorphisms (SNPs) with disease severity. Confirmation of these novel SNPs in a different cohort is required.

Overall, we consider that data demonstrating that the hyperinflammatory phenotype is associated with severe dengue, and that the phenotype is associated with underlying dysfunction of cellular immunity and specific NK cell SNPs is sufficient to move forward with a trial of immunomodulation ^19, 22^. 

We plan to investigate genetic signatures in specific immune cell subsets over the disease course in the trial patients, assessing differences with clinical outcomes and IL-1R blockade. We will assess the gene expression profile from NK and CD8+ T cells (which will address the confounding influences of unseparated cells) at two time-points: enrolment and day 5 following anakinra or placebo. Transcriptomic profiling will provide further understanding of the pathogenic pathways in patients with dengue and the effect of therapeutic IL-1R blockade on these pathways.

### Hypothesis and aims of the trial

Our overarching hypothesis is that IL-1 mediated pathways drive hyperinflammation and severe disease in dengue. We hypothesize that therapeutic IL-1R blockade in selected patients with dengue will improve clinical outcomes through attenuation of the inflammatory responses.

This phase II randomized controlled trial primarily aims to assess the impact of anakinra on clinical outcomes in dengue patients with hyperinflammation. The accompanying immunological and transcriptomic analyses aim to identify novel mechanisms and pathways that may represent future biomarkers and therapeutic targets.

## Methods

### Design

**Figure 1.**
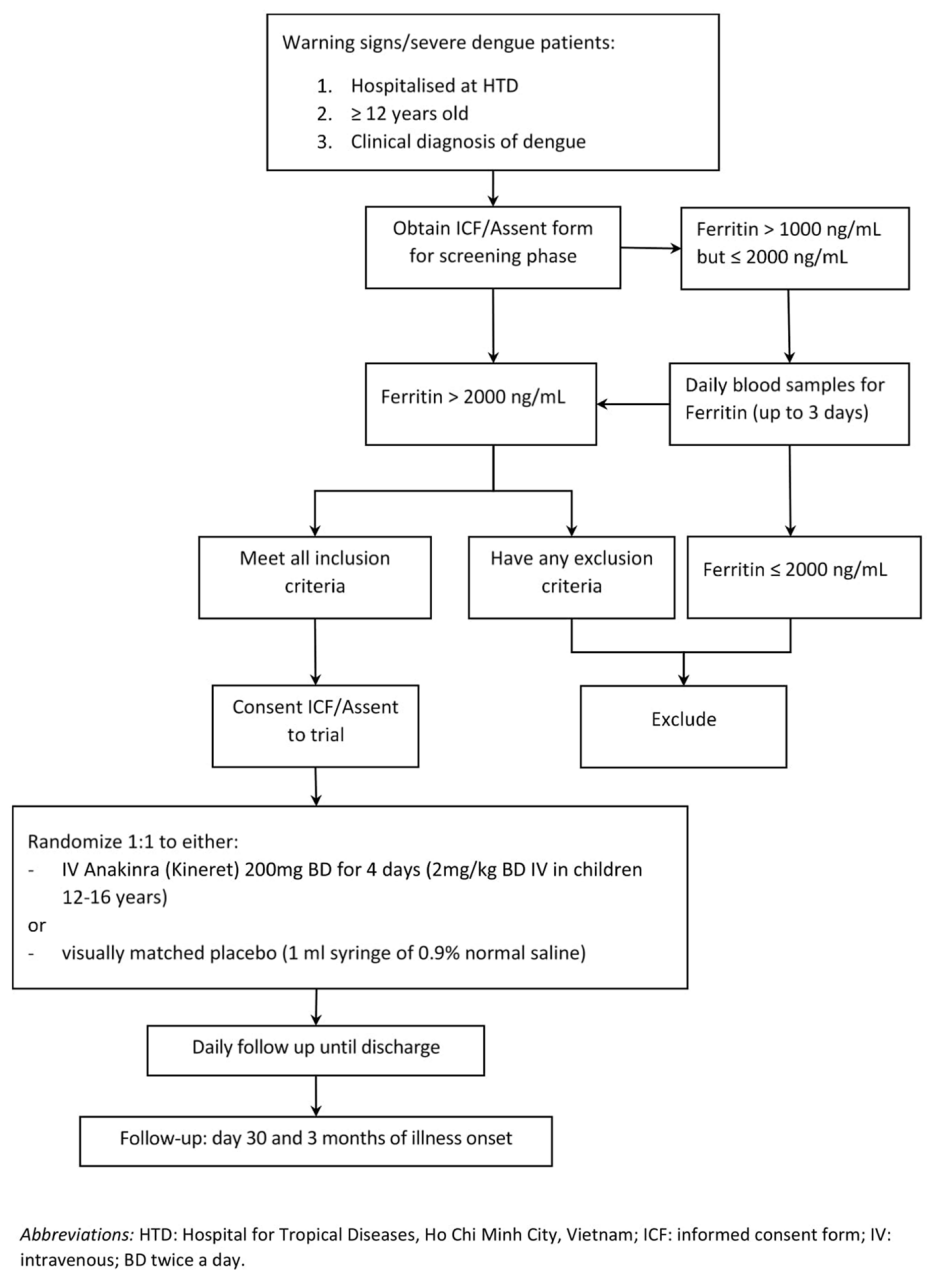
Trial flowchart.

This is a double-blinded randomized placebo-controlled phase II trial of anakinra in patients diagnosed as having dengue with warning signs/severe dengue and hyperferritinaemia (>2000 ng/ml) ( Figure 1). The study will enrol 160 adults and children ≥ 12 years old admitted to the Hospital for Tropical Diseases, Ho Chi Minh City. Participants will be randomized 1:1 (anakinra:placebo) on electronic database system (Ennov) and stratified according to the ward of recruitment (Intensive Care Units/Emergency Department or the general dengue wards). Patients with warning signs or severe dengue (defined by 2009 WHO classification) and ferritin > 2000 ng/ml will be eligible for enrolment.

Intravenous (IV) anakinra (200 mg twice daily) or visually matched placebo (0.9 % normal saline) will be given for 4 days.

### Inclusion and exclusion criteria

Patients will be eligible for study enrolment if they: (i) are more than or equal to 12 years of age; (ii) have a clinical diagnosis of dengue and at least one warning sign or severe dengue (based on the World Health Organization (WHO) 2009 or Vietnamese Ministry Of Health (MOH) – Dengue guidelines) ^23, 24^; (iii) have plasma ferritin levels > 2000 ng/mL ; (iv) agree to come back for two follow up visits between day 30 – 35 and at around day 90 afterillness onset; and (v) provide written informed consent.

Exclusion criteria include: (i) pregnancy (confirmed by either clinical examination or urine dipstick for human chorionic gonadotropin hormone); (ii) localizing features suggestive of an alternative diagnosis; (iii) taking immunosuppressive drugs or other biologics in the last month; (iv) underlying malignancy or immunosuppression; (v) children less than 12 years of age; (vi) have baseline GFR < 30 ml/min; (vii) having a neutrophil count < 1.0 × 10 ^9^/L; (viii) being treated for tuberculosis; (ix) HBsAg-positive; (x) taking any drug with significant interaction with anakinra (concomitant medication and drug interactions); (xi) patients who are considered unlikely to attend follow-up visits because of long travelling distance to the study clinic or other reasons.

### Primary endpoint

The primary endpoint of this study is the delta mSOFA score* (change in mSOFA score over 4 days after randomization).

*mSOFA = modified Sequential Organ Failure Assessment score (modified for resource limited settings and dengue) see Appendix 1 for score.

### Secondary endpoints

The secondary endpoints are: 30-day mortality; change in mSOFA score at day 7 post randomization; number of days in ICU; number of days in hospital; number of individuals with serious adverse events (time-period 1–5 days and 6–30 days); number of adverse events per individual during the whole study course; change in ALT and platelets over 5 days following randomization; change in ferritin and CRP levels over 5 days following randomization and until day 30 after illness onset; time to normalization of platelets (defined as >150 ×10 ^9^/l) and neutrophils (>2 ×10 ^9^/l); platelet nadir; fever clearance time (defined as <37.5 °C for at least 48 hours; measured 4 times/day); duration of viraemia (number of days to first negative qPCR and NS1); AUC viraemia (from enrolment to negative); EQ-5D-5L questionnaire score at hospital discharge and day 30.

### Exploratory endpoints

- T/NK cell phenotype/activation over 5 days following randomization, 1 month and 3 months
- Immune cell gene signatures over 5 days following randomization and day 30
- Differential gene expression over 5 days following randomization and day 30

### Screening

Patients hospitalized to one of the study wards of the Hospital for Tropical Diseases (HTD), Ho Chi Minh City, Vietnam with clinically suspected dengue with warning signs/severe dengue will be identified and, if potentially eligible, approached by study staff. If patients agree to participate in the trial and provide the written consent/assent form, they will be screened for eligibility based on the criteria listed in the section above. Initial screening tests include baseline ferritin, creatinine, HBsAg and, in all female patients, a pregnancy test using a urine dipstick.

Potentially eligible patients who have baseline ferritin levels > 1000 ng/ml but ≤ 2000 ng/ml on admission will be invited to continue the screening phase of the trial which lasts for 3 days with daily ferritin testing. If the ferritin level rises to above 2000 ng/ml during this period, and all inclusion criteria are met, the patient will be recruited. If the ferritin remains below the 2000ng/ml threshold after 3 days, the screening phase will end and all study procedures will stop. Patients will also be excluded if their ferritin levels are below 1000ng/ml.

### Randomization

After confirmation of eligibility, including obtaining ICF for randomization phase, study doctors will perform randomization on the electronic data system (Ennov). Randomization will be minimization at 1:1 ratio and stratified according to the ward of recruitment. The system will assign a unique study ID and drug code for the patient. The assigned drug code will correspond to a sealed pre-packaged box containing 08 doses (bd dosing for 4 days) of either anakinra or visually matched placebo for adults or children. Drug appearance and administration schedules will be identical to maintain blinding amongst the attending physicians and nurses. Neither the patients nor the study assessors, including treating physicians, study doctors/nurses, know which group patients in.

### Study treatment, monitoring and follow-ups

Subsequent study procedures includes: daily study drug administration, daily clinical assessment, case Record Form (CRF) completion and blood sampling for a full blood count, biochemistry including ferritin, diagnostics, other inflammatory and vascular biomarkers and RNA extraction.

Participants will receive study treatment with Anakinra (Kineret) or visually matched placebo, given intravenously 200mg twice a day for 4 days (or 2mg/kg IV BD in children). Other general management of the febrile episode will be at the discretion of the treating physicians.

Study drugs will be stored in a refrigerator in a secure location at 2°C to 8°C and protected from light. All medication storage and administration will be regulated through the central pharmacy of the hospital to ensure good quality and control of medication handling. For the purpose of ambulatory use, Kineret may be kept at room temperature up to 25°C for a maximum of 72 hours.

All patients will be asked to come back for follow-up visits at day 30 and day 90 after fever onset. At these times, research blood samples will be taken for convalescent serology, immunology studies, a full blood count and biochemistry tests. Patients will be contacted 3 – 5 days prior to the follow-up visit to arrange an appropriate appointment date and time. Any patient who misses the follow-up visit will be contacted by phone either to reschedule the visit or to collect information if patients are unable to return in-person. 

### Unblinding

The Clinical Trials Pharmacist will hold the unblinded randomization list with details of the contents of each box of syringes. This list will be accessed only in the case of emergency unblinding, authorized by an investigator as per standard operating procedures. Emergency unblinding will only be performed in the case of an adverse event when the knowledge of the identity of the treatment will contribute to the treating physician’s ability to care for the patient.

### Safety review and monitoring

The trial will be conducted in two phases. The initial pilot phase (cohort 1) includes 10 adults (≥ 16 years old) who will be recruited and monitored closely sequentially during the study course. Each patient will be administered either anakinra or placebo for 4 days (5 anakinra, 5 placebo).

A Data Monitoring Committee (DMC) review will take place when day 5 data are available from the first 10 patients enrolled. Based on a review of all reported serious adverse events and overall clinical outcomes, the DMC will recommend continuation, amendment of the protocol or stopping the study. Following satisfactory safety review after the 10 ^th^ patient, the study will advance to the next phase to enrol the remaining 150 patients. The DMC will continue performing interim reviews after every 50 recruited patients to evaluate safety and tolerability of the study drug. In the event that any unusual or unpredictable serious adverse event occurs, the study team will temporarily pause recruitment and report data to local ethics committees for review.

The study drug will be stopped for an individual if they (1) request drug discontinuation ; (2) develop any AEs which require discontinuation of the study medication, including severe hypersensitivity reaction, severe acute renal impairment (eGFR below 30mL/min/1.73m ^2^), severe neutropenia (absolute neutrophil count < 0.5 × 10 ^9^/L for at least 2 consecutive days [24 ± 6 hours]).

All AEs occurring during the trial will be recorded in the CRF regardless of their relativity with trial medication and will be reported to OxTREC in the annual review form, to the DMC in accordance with the DMC charter and to local ECs following local regulations. Clinical and Laboratory events will be graded according to Common Terminology Criteria for Adverse Events (CTCAE) definitions.

In cases of adverse events leading to i) early stopping of the study drug; or ii) patients having to stay in hospital for longer than 5 days, the treatment for these cases will be at discrete of the attending physicians. If study drug discontinued early due to adverse events, participants will be followed up until the events have resolved for stabilized. Patients who wish to continue to stay in the trial will be followed-up as per protocol. The blood samples and data required in the study will continue to be collected until study day 5. The summary of clinical outcome will be collected at discharge. All patients will be invited to come back for the final follow-up visit at around day 30 of illness and at 3 months.

### Data collection

Study data will be collected using an electronic CRF, except identification information which will only be recorded on paper CRF. All patients will be reviewed daily until discharge. Standardized clinical information and a full blood count, biochemistry and research blood sampling will be performed at each visit, plus any other tests deemed necessary by the attending physician. Details of all adverse events will be recorded on specific forms. An ultrasound scan of the chest and abdomen to assess vascular leakage will also be performed on alternate days in all participants by a study staff member using the portable ultrasound scan machine provided by OUCRU.

During the follow-up visits at day 30 and 3-months after the fever onset, patients will be asked about any newly emerging or persisting symptoms using a standardized questionnaire. Research blood samples will be taken for convalescent serology, immunology studies, full blood count and biochemistry tests.

### Statistical considerations

***Sample size.*** Given that delta mSOFA score has an approximately normal distribution ^25^ with a standard deviation of 2.4, which was based on a cohort of 38 adults admitted with severe dengue, we would need a sample size of 80 patients per group to detect a between-group difference of at least 1.0 point with 80% power and 5% type-I error rate.

***Statistical analysis.*** The primary endpoint (delta mSOFA score) and the secondary endpoints (ALT, CRP, PLTs and ferritin) will be analyzed using a linear mixed-effects model, which models their values over the first 4 days for mSOFA and 5 days for lab tests (as specified in sections 2.4.1 and 2.4.2). We will use a linear trend for time as fixed and random effects. The slope of this model reflects the change in value over time. Therefore, the effect of anakinra is measured as the difference in slope between treatment groups. This difference will be assessed via the interaction between time and treatment group in that model, and presented with a corresponding 95% confidence interval. For the change at day 30 (ferritin and CRP) we will use the difference with the value at randomisation. Mortality will be assessed by comparing Kaplan-Meier curves via log-rank test. The number of serious adverse events will be compared via 2×2 tables with chi-square tests, or Fisher’s exact test in case the expected number under the null hypothesis is smaller than 1 in at least one of the cells. Number of AEs per individual will be compared using Poisson regression, or quasi-Poisson regression in case of overdispersion. EQ visual analogue score will be compared via linear regression in case it has a fairly symmetric distribution; otherwise we will use beta-regression. Normalization of neutrophils will be defined as first time to reach normal range of >2.0 ×10 ^9^/l and for platelets the first time to reach >150 ×10 ^9^/l and will be assessed by Cox model. For viraemia AUC and duration outcomes we will also perform the analysis with correction for day of illness (DOI). The time of fever clearance is defined by the first time after which all readings are <37.5°C in the following 48 hours.

## Ethical considerations

### Ethical approval

The current protocol version 4.0 dated 07 DEC 23 and relevant documents (ICFs, assent forms, and participant information sheet) have been approved by the Ethics Committee of Hospital for Tropical Diseases, Ho Chi Minh City, the Vietnam Ministry of Health Ethics Committee and the Oxford Tropical Research Committee i.e. OxTREC.

The Investigator will obtain approval from the above parties for all amendments to the approved documents, where necessary.

### ICF and PIS

Prior to any study procedures, a written ICF will be obtained from eligible patients who agree to participate. If a patient is in a condition that prevents them from making informed decisions, the informed consent may be obtained from a legal representative. Once their condition has improved, study staff will explain the study to the patient and ask them to counter-sign the ICF if they agree to continue the study. Patients between 12 and 18 years of age will be asked to sign the assent form together with the ICF signed by their parents/guardian. All patient information sheets (PIS) and ICFs will be written in the local language and will use terms that are easily understandable.

During the informed consent process, the following will be explained by a study physician: the study purpose, procedures, possible risks/benefits, the rights and responsibilities of participants and alternatives to enrolment. Potential participants will be encouraged to ask questions and provided with a study contact number should they have any subsequent queries. In addition to the procedures above, illiterate signatories will have the ICF read to them in the presence of a witness who will sign to confirm this.

### Data management

All study data will be recorded on standard electronic CRFs at the study sites. All data management activities will be carried out to ensure data quality and confidentiality.

Participants will be assured that all information generated in this study will remain confidential. CRFs and samples will be labelled with a study identification number only and stored in suitable secure locations. All study data will be stored in password-protected databases, to which access is only limited to responsible study staff and authorised representatives from the University of Oxford and any host institution for monitoring and/or audit of the study to ensure compliance with regulations. Participants’ names will be recorded at the time of enrolment to allow for their identification at follow-up visits, however, identifiable information will be linked to stored data or samples only by a protected Master List. Any scientific publications or reports will not identify any patient by name or initials. When the research team reviews the clinical notes, they will also be bound by professional confidentiality.

Following conclusion of the project, data will be stored in a safe place. Original paper documents will be stored at the OUCRU storage for 15 years after which paper files will be scanned and archived electronically according to OUCRU SOPs. Electronic data will be stored indefinitely on a secure OUCRU-VN server for a minimum period of 10 years and paper copies will be destroyed securely.

### Data monitoring and trial steering committees

Data monitoring will be carried out by OUCRU CTU staff. The frequency, type and intensity of routine monitoring and the requirements for triggered monitoring will be detailed in the Monitoring Plan which will also detail the procedures for review and sign-off. The monitoring will adhere to the principles of ICH GCP and the Monitoring Plan.

An independent Data Monitoring Committee (DMC) consisting of qualified volunteers, with the necessary knowledge of clinical trials and statistics, will review the protocol and agree to a data review schedule and reporting requirements before the study commences. All data reviewed by the DMC will be in the strictest confidence. An analysis of overall clinical outcome will be performed. The report from the DMC will be forwarded to the local ethics committees for their review.

A Trial Steering Committee (TSC) will provide overall supervision of the conduct of the trial and provide advice through its independent Chair. The ultimate decision for the continuation of the trial lies with the TSC. In particular, the TSC will concentrate on progress of the trial, adherence to the protocol, patient safety, and the consideration of new information.

## Data sharing and dissemination

In line with research transparency and greater access to data from trials, this study has been registered at ClinicalTrials.gov ( NCT05611710) on 19 Oct 2022 and a data sharing policy is in place. Data exchange complies with Information Governance and Data Security policies in Vietnam and the UK.

Participants will not be individually informed of results. Data from this study will be published in peer-reviewed journals. References guidelines and all authors will have made a noteworthy contribution to the work.

## Trial status

The trial is ongoing. Recruitment started in January 2024.

## Discussion

Dengue remains a major global public health burden. Over the last few decades, the geographic distribution of the Aedes mosquito vector has been expanding and dengue cases have been increasing; the increasing incidence is predicted to continue with ongoing urbanization, demographic shifts and climate change ^26, 27^. Despite the increasing burden, there are currently no approved treatments to either reduce viral replication or to modify the deleterious host inflammatory response to infection. Development and investigation of novel therapeutics has been slow due to disinterest in this historically neglected tropical disease. 

Like other acute viral infections, including Ebola, SARS-CoV2 and influenza, severe dengue is associated with an excessive host inflammatory response presenting in the critical phase of disease (usually days 4–6 of infection). High IL-1, ferritin, and C-reactive protein are all associated with severe dengue ^15, 28– 30^. In particular, ferritin has emerged as a useful prognostic biomarker; higher ferritin is associated with increased risk of disease progression, higher SOFA score, need for intensive care admission and mortality ^16, 28^.

Until recently, the mechanistic basis for hyperferritinaemia and hyperinflammation in dengue hasn’t been well understood, but recent insights suggest that it occurs due to a combination of unchecked viral replication within macrophages and monocytes, with viral uptake mediated by non-neutralising antibodies from prior infection with a heterotypic dengue serotype, in addition to dysfunctional NK T cells with impaired cytolytic function with reduced perforin and granzyme B production ^19^.

As an IL-1 receptor antagonist, Anakinra targets the IL-1β hyperinflammatory loop which mediates hyperferritinaemia, coagulopathy, fever and tissue damage. Anakinra improved survival in a subgroup of patients with sepsis-associated hyperinflammation (ferritin >2000 ng/mL, coagulopathy, and liver enzyme elevations) ^31^. Other broadly acting immunosuppressives such as corticosteroids or more targeted modulation with IL-6 inhibitors (tocilizumab) and Janus-kinase (JAK) inhibitors (baricitinib) have now become standard of care for managing hyperinflammation in severe COVID-19. In light of the growing evidence base that immune modulation can improve outcomes in patients with an excessive host immune response to infection, we believe that anakinra is a promising treatment for the IL-1 mediated cytokine storm which drives hyperinflammation in dengue. This currently licensed drug has a promising safety profile, wide therapeutic indications, a short half-life and can be given intravenously, ensuring effective delivery in patients unable to take or absorb oral medications.

Trials of immune modulation in COVID-19 and sepsis have cemented the theory that identifying and targeting the correct subpopulation of patients is key to both finding those most likely to benefit from immunomodulation, and minimising harm to patients who would be unlikely to benefit. Accordingly, this will be the first trial of immunomodulation in dengue using biomarker-guided enrichment for patient selection.

## Ethics and consent

The protocol and relevant documents (ICFs, assent forms, and participant information sheet) have been approved by the Ethics Committee of Hospital for Tropical Diseases, Ho Chi Minh City (decions no 3316/QÐ-BVBNÐ dated 18 OCT 22), the Vietnam Ministry of Health Ethics Committee (decision no 3381/QÐ- BYT dated 30 AUG 23) and the Oxford Tropical Research Committee i.e. OxTREC (dated 03 JAN 23 , OxTREC reference 17–22). Participants will provide written informed consent.

## Data Availability

No data are associated with this article. Figshare: Appendix 1 (mSOFA) for ‘Anakinra for dengue patients with hyperinflammation protocol for a randomized double-blind placebo-controlled. https://doi.org/10.6084/m9.figshare.27209823.v1 ^32^ Figshare: SPIRIT checklist for ‘Anakinra for dengue patients with hyperinflammation: protocol for a randomized double-blind placebo-controlled trial’. https://doi.org/10.6084/m9.figshare.27159879 ^33^. Data are available under the terms of the Creative Commons Zero "No rights reserved" data waiver (CC0 1.0 Public domain dedication).
