# Supplementary material for: Anakinra for dengue patients with hyperinflammation: protocol for a randomized double-blind placebo-controlled trial
Source: Wellcome Open Res. 2024 Nov 22;9:689. [Version 1] doi: 10.12688/wellcomeopenres.21017.1 (PMC11809184; doi:10.12688/wellcomeopenres.21017.1)
Supplement: Peer review history

# Reviewer report: Reviewer response for version 1

**DOI:** 10.21956/wellcomeopenres.23252.r114083

**Published:** 2024-12-20

**Authors:** Bachti Alisjahbana

**Affiliations:** 1 Universitas Padjadjaran, Bandung, West Java, Indonesia

This is  very nice and interesting study, yet quite challenging task. Currently we do not have any treatment which can alleviate or prevent the complication of severe dengue.  In this case, a novel host-directed therapies are urgently needed for patients with dengue. Many studies have been conducted including the use of immunosuppression using corticosteroids but so far been unsuccessful.

 Severe dengue is marked by increased inflammatory marker, including TNF alfa, IL-1 etc. Some studies have shown that the increased of these inflammatory markers are related to plasma leakage and shock. It is therefore a rational thought to see if the blocking of these cytokines can alleviate the bad pathology of severe dengue. This study is aimed to test if Anakinra, a IL1R antagonist has the efficacy to alleviate the complication of severe dengue.

 The investigator plans to include 160 patient with high inflammatory condition, marked by Ferritin level > 2000. These Patient will be Randomized to acquire Anakinra or placebo, for 4 consecutive days. To measure the efficacy of anakinra the change in mSOFA score over 4 days after randomization will be evaluated. Secondly, the accompanying immunological and transcriptomic markers will be analyze to identify novel mechanisms and pathways that may represent future biomarkers and therapeutic targets.

 This study is novel, it has the potential to find new process or Pathological Mechanism of severe dengue. However, limitations exist. Due that Dengue is an acute process, often the initial process in the first three days cannot be distinguish if we receive patient at the late stage of the disease.

 Therefore I would like to posed several attentions:

 Day of fever at inclusion. In the study there is no clear at which day of fever  the investigator expect the patient to be included. Most dengue patient in the tropics, comes at day 4 to 6. Would this be adequate for the anakinra to have an effect. Previous report have shown that immunosuppressive have some effect if given at the beginning of the fever like at the first 1-3 days.  

 And secondly, when is the latest day of fever that we  think it is not useful to include patient? If a patient comes at day 8 or 9 of fever? Because dengue patient who are still ill at day > 8 because of dengue fever may also have secondary bacterial infection as their complication. This may confound some of the results.  

 Perhaps it would be necessary to foresee how long it will take to include these 160 subjects. If the study is conducted for too long, it may lose its significancy and become less ethical to be conducted. To estimate the time of enrollment, I think the investigator should have some secondary data on how many Dengue patients having ferritin > 2000 in the last one year in the Designated Hospital.

Is the study design appropriate for the research question?

Yes

Is the rationale for, and objectives of, the study clearly described?

Yes

Are sufficient details of the methods provided to allow replication by others?

Partly

Are the datasets clearly presented in a useable and accessible format?

Not applicable

Reviewer Expertise:

Internal Medicine, Infectious Diseases, Immunology. Especially in disease group of Tuberculosis, Dengue, Typhoid, and Sepsis.

I confirm that I have read this submission and believe that I have an appropriate level of expertise to confirm that it is of an acceptable scientific standard.

# Reviewer report: Reviewer response for version 1

**DOI:** 10.21956/wellcomeopenres.23252.r113221

**Published:** 2024-12-19

**Authors:** Panisadee Avirutnan, Dumrong Mairiang

**Affiliations:** 1 Division of Dengue Hemorrhagic Fever Research & Siriraj Center of Research Excellence in Dengue and Emerging Pathogens, Mahidol University, Bangkok, Thailand; 2 Medical Biotechnology Research Unit, National Center for Genetic Engineering and Biotechnology, Siriraj Hospital, Bangkok, Thailand

Huyen et al. present a protocol for a randomized, placebo-controlled phase II trial investigating the efficacy of Anakinra, an IL-1 receptor antagonist, in treating severe dengue patients aged 12 years and older. The study will be conducted at the Hospital for Tropical Diseases in Ho Chi Minh City, Vietnam, and will include patients with dengue with warning signs or severe dengue. The primary objective is to assess the reduction in disease severity, measured by the change in modified Sequential Organ Failure Assessment (mSOFA) score over four days following randomization. Ferritin levels will be used as a screening tool to predict severe disease progression. While the rationale and objectives are clearly presented, and the methods are generally well-described, the study design raises several concerns, particularly regarding the sample size calculation, the generalizability of the primary outcome measure across different age groups, and the potential impact of combining pediatric and adult populations in the analysis. 

**1.    Sample size calculation**

 The authors propose enrolling 160 patients (80 per arm) to detect a 1.0 mSOFA point difference with a standard deviation of 2.4 points, using 80% power and a 5% significance level. However, calculations using standard power analysis methods for comparing two means (Rosner, 2015, Fundamentals of Biostatistics. 8th ed. USA: Cengage Learning) indicate that a minimum of 91 patients per arm (182 total) would be required to achieve the desired power.

 To address this discrepancy, the authors should:

 •    Provide a detailed explanation of their sample size calculation, including the specific formula or software used. This will allow for independent verification of their calculations and ensure transparency.

 •    Address the potential impact of participant dropout or death on the study's power. Given the severity of dengue, it is likely that some participants may withdraw or die before completing the study. The authors should stimate the anticipated dropout rate based on previous studies or clinical experience and adjust the sample size accordingly to ensure sufficient power even with attrition. This could involve increasing the initial enrollment or implementing a clear plan for replacing participants who are lost to follow-up. For example, if a 20% dropout rate is anticipated, enrolling at least 200 patients would be necessary to ensure that at least 160 complete the study. A clear strategy for handling missing data should also be outlined in the protocol.

**2.    Generalizability of mSOFA scores**

 The sample size calculation relies on data from a previous study (Ref #25) conducted exclusively on adult severe dengue patients. However, this protocol includes both pediatric patients and those with dengue with warning signs, raising concerns about the generalizability of the mSOFA score data.

 Specifically:

 •    Pediatric patients may exhibit different mSOFA score patterns compared to adults. Physiological differences and variations in disease manifestation could influence the mSOFA score, potentially affecting the study's power. The authors need to provide evidence supporting the applicability of the previous study's mSOFA score data to the pediatric population or adjust the sample size accordingly.

 •    While ferritin levels will be used to predict severe dengue progression in patients with warning signs, it remains unclear how mSOFA scores will behave in those with elevated ferritin (>2000 ng/ml) who do not progress to severe dengue. This subgroup could introduce variability in mSOFA scores, potentially impacting the study's power. The authors should address this potential heterogeneity and its implications for the sample size calculation.

 It is crucial to ensure that the chosen sample size adequately accounts for the potential variability in mSOFA scores across the different patient subgroups to maintain the study's power and validity.

**3. Age stratification and ward-related confounding**

 Pediatric and adult patients are generally treated in different wards with different sets of medical staff, potentially introducing confounding factors that could influence treatment outcomes. These factors may include variations in clinical practices (e.g., treatment protocols, monitoring practices, supportive care) and ward environment (e.g., facilities, resources, nurse-to-patient ratios). Such differences could influence the course of dengue and the response to Anakinra, potentially obscuring the true treatment effect.

 The authors propose a phased approach, starting with a small cohort of adult patients to assess safety before enrolling children. This is commendable from an ethical standpoint, as it prioritizes the safety of children and allows for preliminary assessment of potential adverse events before including this vulnerable population.

 However, the concerns regarding the generalizability of mSOFA scores and the potential differences in disease manifestation and treatment regimes between adults and children remain significant. To mitigate these concerns, the following is recommended:

- Stratify participants by age group (e.g., 12-15 versus >15) to ensure that each treatment arm has a similar proportion of pediatric and adult patients. This will help to minimize the influence of ward-related factors on treatment comparisons.
- Conduct a sub-analysis to evaluate the potential impact of ward-related confounding factors on treatment outcomes. This will help to determine whether any observed differences in efficacy are truly attributable to Anakinra or if they are influenced by variations in care between pediatric and adult wards.

**4.    Timing of anakinra administration**

 The timing of Anakinra administration is critical in dengue management. Early administration during the initial febrile phase may interfere with the necessary immune response, potentially leading to prolonged viremia or increased risk of complications. Conversely, late administration, when severe complications have already manifested, may render the treatment ineffective in mitigating organ damage and systemic inflammation.

 Given that the authors use ferritin levels as an inclusion criterion, it is essential to consider the dynamic nature of ferritin in dengue.  Using a single ferritin threshold for inclusion may not accurately identify patients at the optimal time for Anakinra intervention. 

 To optimize the therapeutic potential of Anakinra while minimizing potential harm, the authors should:

 •    Clearly define the criteria for initiating treatment. This should include specific clinical and laboratory parameters, including dynamic changes in ferritin levels, that indicate the transition from the early febrile phase to the critical phase where the risk of severe complications is highest.

 •    Provide a detailed rationale for the chosen timing in relation to ferritin levels. The authors should justify their approach based on the current understanding of dengue pathophysiology, the kinetics of IL-1 signaling, and the temporal relationship between ferritin levels and disease severity.

Is the study design appropriate for the research question?

Partly

Is the rationale for, and objectives of, the study clearly described?

Yes

Are sufficient details of the methods provided to allow replication by others?

Yes

Are the datasets clearly presented in a useable and accessible format?

Not applicable

Reviewer Expertise:

dengue immunology, dengue clinical research

We confirm that we have read this submission and believe that we have an appropriate level of expertise to confirm that it is of an acceptable scientific standard, however we have significant reservations, as outlined above.

# Reviewer report: Reviewer response for version 1

**DOI:** 10.21956/wellcomeopenres.23252.r113224

**Published:** 2024-12-16

**Authors:** Gathsaurie Neelika Malavige

**Affiliations:** 1 University of Sri Jayewardenepura, Nugegoda, Sri Lanka

This trial, which aims to study the efficacy of anakinra for reducing disease severity in patients with severe dengue, is a very timely trial given that there are hardly any drugs being previously trialed in patients with severe dengue and hyperinflammation.

 The trial is planned in such a manner that it will also capture the immune responses and differential gene expression over time in patients with severe disease, which has not been studied before. Therefore, even if this trial gives a negative outcome by any chance (not meeting the trial endpoints), an enormous wealth of information is likely to be generated from this study that would enable us to understand critical questions related to pathogenesis of severe dengue. Further, it will also provide useful clues regarding potential biomarkers that are associated with severe dengue.

 The data collection, measurements and follow-up has been well planned and thought out carefully. In the inclusion criteria, although its not clear, I believe the participants will only be recruited if all five criteria are full filled?

Is the study design appropriate for the research question?

Yes

Is the rationale for, and objectives of, the study clearly described?

Yes

Are sufficient details of the methods provided to allow replication by others?

Yes

Are the datasets clearly presented in a useable and accessible format?

Yes

Reviewer Expertise:

I have been studying dengue pathogenesis for around 15 years and have been working on T cells, inflammatory mediators, antibodies and vascular leak on dengue. I have been involved in identifying potential drug targets and leading two clinical trials on dengue.

I confirm that I have read this submission and believe that I have an appropriate level of expertise to confirm that it is of an acceptable scientific standard.

# Reviewer report: Reviewer response for version 1

**DOI:** 10.21956/wellcomeopenres.23252.r114078

**Published:** 2024-12-04

**Authors:** Arif Nur Muhammad Ansori

**Affiliations:** 1 Universitas Airlangga, Surabaya, East Java, Indonesia

First of all, this manuscript is good and well-written. However, I may require some comments on the following issues. Basically, this manuscript is acceptable.

 However, in order to improve the readability of the paper by an English editing service as various sentences are mistyped. 

**Title: **The title is easy to follow and no mistakes.

**Abstract: **This section was well-written and easy to understand. In addition, the study's objective and the state of the art of the study is clear. Furthermore, the keywords should represent the study.

**Discussion: **The discussion of the study is lack of information about the important topics. To develop a systematic discussion, the authors should mention the limitation(s) of the study.

**Conclusion: **In this part of the paper, the authors have established the conclusion of this study.

 References: Many references are out of guidelines. Please kindly check the references again, do not include the old references, and check the abbreviation of the journals/proceedings. Please mention many high-quality related publications in 2024.

Is the study design appropriate for the research question?

Yes

Is the rationale for, and objectives of, the study clearly described?

Yes

Are sufficient details of the methods provided to allow replication by others?

Yes

Are the datasets clearly presented in a useable and accessible format?

Yes

Reviewer Expertise:

Virology and Immunology

I confirm that I have read this submission and believe that I have an appropriate level of expertise to confirm that it is of an acceptable scientific standard, however I have significant reservations, as outlined above.
